# National HTA system in Canada: From system analysis and understanding to system strengthening

**DOI:** 10.1371/journal.pgph.0005858

**Published:** 2026-04-29

**Authors:** Mohammed Alkhaldi, Rima Kachach, Aisha Al Basuoni, Line Enjalbert, Malak Alrubaie, Maya Hassan, Sara Ahmed

**Affiliations:** 1 Department of Public Health, School of Health Sciences and Psychology, Canadian University Dubai, Dubai, United Arab Emirates; 2 Faculty of Medicine and Health Sciences, School of Physical and Occupational Therapy, McGill University, Montreal, Canada; 3 Centre for Outcomes Research and Evaluation (CORE), McGill University Health Center, Montreal, Canada; 4 Centre for Interdisciplinary Research in Rehabilitation of Greater Montreal (CRIR), The Integrated University Health and Social Services Centre of West-Central Montreal (CIUSSS West-Central Montreal), Center for Outcomes Research and Evaluation, Clinical Epidemiology, Montreal, Canada; 5 Centre for Tropical Medicine and Global Health, Nuffield Department of Medicine, University of Oxford, Oxford, United Kingdom; 6 Faculty of Health Sciences, American University of Beirut, Beirut, Lebanon; 7 Projects Unit, Gaza Community Mental Health Programme, Gaza, Palestine; 8 Faculty of Health, University of Waterloo, Waterloo, Ontario, Canada; National Center for Chronic and Noncommunicable Disease Control and Prevention, Chinese Center for Disease Control and Prevention, CHINA

## Abstract

Health Technology Assessment (HTA) has played a critical role in guiding evidence-based decision-making in Canada’s healthcare system; however, its current structure and processes face several limitations that reduce its effectiveness and responsiveness. To effectively address these challenges, there is a clear need for a comprehensive framework that outlines the key components of Canada’s HTA system—including its conceptual foundations, policy environment, structural organization, governance models, institutional capacities, implementation pathways, and integration into decision-making processes. Such a framework would support more coordinated, transparent, and responsive HTA practices across jurisdictions. Using a systems thinking and analytical approach, the study aims to comprehensively analyze the components of the national HTA system in Canada. The study was conducted between 3^rd^ May 2021 and 22^nd^ April 2022, selectively recruiting experts and organizations involved in HTA. Seven national HTA experts participated in virtual In-Depth Interviews (IDIs) to understand HTA from a policy perspective, and ten HTA-associated organizations from the Canadian governmental and non-governmental sectors completed one electronic institutional survey to understand HTA from a technical perspective. Findings indicate a high level of literacy/understanding and perceived applicability of HTA, though challenges exist in governance, legislation, and decentralized coordination. While there’s general support for HTA, concerns arise regarding the extent of HTA report utilization and the need for a more cohesive national HTA approach. Capacity-wise, sustainable funding exists, but challenges include varying assessment coverage and the lack of harmonized guidelines resulting from a decentralized structure revealed within provinces. Qualitative findings revealed a decentralized structure with provincial-level HTA bodies, limited national coordination, and inconsistent integration into decision-making. Experts emphasized the need for standardized guidelines, improved capacity, and stronger national oversight to enhance the utilization of HTA. This study proposes advancing national coordination and developing standardized HTA guidelines to address fragmentation. It also recommends investing in capacity-building, training, and sustainable funding to strengthen HTA implementation. In conclusion, this study generates evidence of the strengths of the Canadian HTA system, as well as the presence of significant legislative, structural, policy, and capacity-related challenges. Despite the available funding, human resources, and capacity, and the active role of the Canadian Agency for Drugs and Technologies in Health (CADTH), establishing national HTA frameworks and strategies is a priority. This strategy, along with reinforcing provincial and federal stakeholder engagement, improving adoption pathways of HTA recommendations, and adapting HTA methodologies for more flexible and timely evaluations, can strengthen the Canadian HTA system.

## Introduction

Health Technology Assessment (HTA) is a vital process widely employed in high-income countries (HICs) to systematically evaluate healthcare technologies’ safety, efficacy, cost-effectiveness, and broader societal impact [1]. HTA is a multidisciplinary process that uses explicit methods to determine the value of health technology at different points in its life cycle. The purpose is to inform decision-making to promote an equitable, efficient, and high-quality health system [2]. By leveraging evidence-based methodologies, HTA serves as a cornerstone for guiding healthcare decision-making, resource allocation, and policy development, leading to more equitable and efficient healthcare systems [3].

Canada’s healthcare system, like others worldwide, is immersed in a process of development, attempting to adapt conventional frameworks of HTA and funding models to a new landscape of precision-based decisions [4]. This system is decentralized as provinces and territories fund, regulate, and provide health services [4]. Canada provides Universal Health Coverage (UHC) for hospital and physician services but excludes universal insurance for prescription medicines. The federal government oversees the Canada Health Act, which ensures all Canadians have access to hospital and primary healthcare services. Both federal and provincial/territorial governments and their agencies have roles in setting policy and regulating drug prices and costs [5]. Canada’s UHC system, often referred to as Medicare, provides public coverage mainly for hospital (91%) and physician (99%) services. However, Canada has relatively high out-of-pocket expenditure (19% of spending) and is currently the tenth largest pharmaceutical market, following Brazil. It is wrestling with inequitable coverage, low use of biosimilars, and issues of affordability and sustainability, all driven by rare disease drugs [5]. Canada has been ranked 4th in health expenditures per capita among Organization for Economic Co-operation and Development (OECD) countries, and approximately 70% of these expenditures are publicly financed through general taxation by the federal, provincial, and territorial governments [6,7]. In Canada, unequal access to medicines persists across provinces and territories, despite the existence of central, coordinated efforts such as those by the Canadian Agency for Drugs and Technologies in Health **(**CADTH) and the pan-Canadian Pharmaceutical Alliance (pCPA) [8].

In Canada, HTA plays a pivotal role in informing healthcare policies and resource allocation by providing evidence-based evaluations of healthcare technologies. Key organizations involved in HTA include the CADTH and various provincial-level HTA agencies. CADTH, as a national independent and not-for-profit HTA agency, conducts comprehensive HTAs at the federal level. It employs rigorous methodologies to evaluate the clinical efficacy, safety, and cost-effectiveness of technologies, generating evidence-based recommendations to assist federal, provincial, and territorial governments in their decision-making processes [9]. In the Canadian context, “local HTA” typically encompasses hospital- or region-level assessments conducted to support evidence-informed decisions within specific institutions or health authorities, complementing the broader assessments undertaken at the federal or provincial levels. Some challenges faced by local HTA producers to influence hospital policies and clinical practice involve the engagement of healthcare professionals and the potential lack of training and support necessary for the introduction of new technology [10].

In addition to CADTH, Canada’s HTA infrastructure has expanded to provincial and regional levels, fostering alignment of evidence-based information to better support decision-makers. This includes agencies like Quebec’s INESSS and Health Quality Ontario (HQO) as provincial HTA agencies, as well as the Alberta provincial government’s use of partner agencies to produce assessments. Hospital, university, and institutional HTA units also complement these approaches. Increasing collaboration and coordination between all Canadian HTA producers is recognized as a priority [5]. This decentralized approach ensures that healthcare decisions align with the unique healthcare needs and characteristics of different provinces [11].

The clinical and economic value of newly assessed medicines has not always been clear, as only limited information about submissions and final recommendations was publicly available at the time, resulting in reduced transparency [12]. Unlike most HICs with universal public health care, Canada does not include universal access to prescription drug insurance. However, most Canadians regard universal healthcare as one of Canada’s defining qualities and believe ‘access to health care based on need, not ability to pay’ is a core value [5]. Moreover, Canada has no national policy for drugs treating rare diseases (orphan drugs). Nevertheless, in 2022, the Canadian government committed to creating a national strategy to make access to these drugs more consistent. Introducing a national strategy for orphan drugs increases transparency, consistency, collaboration, and access to essential drugs, despite the barriers to the wide adoption of targeted cancer therapies that still exist [13].

Although Canada is a pioneer high-income country in HTA due to its progress, there are still considerable unrecognized challenges related to the national HTA system in Canada. To overcome these challenges, evidence-based solutions generated through an inclusive multi-stakeholder HTA system analysis are required. These solutions enable this system in aspects of awareness, regulation, governance, priority-setting, resources and capacity, implementation, and utilization. The importance of this system thinking and analysis study stems from the paucity of national HTA system analysis research and the limited knowledge of these HTA challenges and aspects. In line with methodologies used in similar studies employing systems thinking and analysis [14–16], the study aimed to enrich the existing knowledge about the HTA system and processes in Canada by conducting a national HTA system analysis to assess the HTA aspects, including understanding, governance, capacity, resources, implementation processes, in addition to barriers, facilitators, and priorities. The findings of this study may guide all stakeholders in developing and strengthening the HTA system in Canada and similar contexts worldwide.

### Research aims and objectives

The overall aim of this study was to develop a comprehensive understanding of the HTA system in Canada and to assess its core pillars through the following specific objectives:

- Assess stakeholders’ level of understanding of the HTA concept, its importance, and related practices;
- Explore HTA stewardship and governance, capacities and resources, and the implementation and use of HTA in health decision-making and policymaking;
- Evaluate how extensively current health technologies, services, and interventions are examined using HTA;
- Identify gaps and propose feasible solutions to support best practices for HTA and knowledge translation strategies at national and regional levels

## Methods

Applying an innovative systems thinking approach, this national HTA system analysis study is part of a larger multi-country HTA systems analysis project that was implemented in various high, middle, and low-income countries, including Canada. This study was conducted between 3^rd^ May 2021 and 22^nd^ April 2022. The study used a cross-sectional design employing mixed methods, including an electronic HTA survey informed by a literature review, and a virtual in-depth interview (IDI), as specified in the study protocol and consistently applied in similar studies [14–17]. Critical and relevant literature included peer-reviewed and non-peer-reviewed articles that captured HTA-related surveys, manuals, guidelines, and frameworks. These were reviewed to adapt the original version of the World Health Organization (WHO) HTA survey. The literature review and survey adaptation/development were conducted by the research team (MA, AAB, and SA), supported by expert consultations. To gather insights into the technical and operational aspects of HTA, the survey was administered to thirteen organizations representing diverse sectors engaged in HTA-related activities. The selection of these organizations was purposive, guided by predefined criteria detailed in the following sections. The following section provides a structured discussion of both data collection tools.

### Electronic institutional HTA survey

The HTA survey was adapted from the WHO global HTA survey [18] enhanced through the conducted literature review and expert consultations with added domains, refined items, and structural improvements to fit the HTA system analysis design. The HTA survey was administered to ten organizations from various sectors involved in HTA to gather information on technical and operational aspects. The survey was completed by designated operational team members, such as HTA officers and staff, on behalf of their respective organizations. This electronic survey consisted of six domains that cover the pillars of the HTA system, with each domain comprising relevant items (questions). These domains include understanding of HTA, the use and application of HTA, implementation of HTA, stewardship and management, resources and capacity, and impediments and insights for strengthening HTA. Further domains and questions of this survey on HTA processes, standardization, and HTA and decision-making were developed based on reviewing recent and relevant literature. The survey questions were closed-ended questions to collect data that reflect the technical, operational, and practical perspectives of HTA aspects. One survey was administered and completed by a member designated by the team in each of the ten organizations involved in HTA. The full survey is enclosed with Supplement 1.

### Virtual in-depth interview

Seven high-level experts representing different sectors and currently engaged in HTA participated in interviews, which focused on policy-related aspects. The IDI guide was developed in accordance with best practices for qualitative research [19], ensuring a rigorous and systematic approach to data collection. Unlike surveys, which gather standardized responses, interviews enable an in-depth exploration of participants’ policy perspectives, strategizing experiences, and decision-making processes. The IDI guide is enclosed in Supplement 2. The guide was designed using semi-structured interview techniques, providing a balance between consistency across interviews and flexibility to adapt to emergent themes. It aligns with established qualitative methodologies [20–22], and questions included are guided by the set of objectives and literature review on HTA. Additionally, the guide adheres to recognized analysis and results reporting standards, such as COREQ (Consolidated Criteria for Reporting Qualitative Research) (enclosed in supplement 3) and SRQR (Standards for Reporting Qualitative Research), enhancing transparency, credibility, and analytical depth in HTA research.

Both tools underwent a rigorous review and consultation process involving ten recognized local and international experts across the fields of public health, health systems, digital technology, health economics, epidemiology, clinical specialties, and health policy and management. Feedback from these experts was incorporated into the final versions of the tools.

### Study population and sampling strategy

Maximum variation purposive sampling [23] was used for this study. The study identified two distinct groups of participants based on specific criteria. The first group consisted of ten HTA-associated organizations, governmental, academic, private, or non-governmental, that operate within the health sector in Canada. Each organization’s management assigned a technical and operational team responsible for HTA processes to complete a single institutional survey on its behalf. The second group comprised experts/leaders from these organizations who are responsible for overseeing HTA policies and strategic issues, and who were selected to participate in individual IDIs. Those experts actively working in HTA decision-making, policy, strategy, and systems were also identified from the same selected HTA-associated organizations.

Two methods were used to explore and identify names and information of main active organizations and individuals in order to achieve maximum variation (experts/leaders): 1) a rapid review of grey and published literature, and 2) extensive consultations among research team members and with collaborators in Canada. These methods helped generate comprehensive lists of organizations for the survey and experts for the IDIs based on predefined criteria and conditions. Our target was to select up to ten existing local and national organizations and up to ten experts from the prepared list. After applying the inclusion criteria, ten major, active, and relevant HTA-associated organizations were selected that met the following conditions: the organization was officially recognized by local and national health authorities, active at least one year since its establishment, had a defined mission for HTA stewardship, production, education, research, and funding; had any previous or current programs, projects, or interventions that directly or indirectly related to HTA; or participated in one or more of HTA activities. The organizations with no direct or indirect role in HTA and those who did not meet the inclusion criteria were excluded. A purposive sampling strategy, conducted in consultation with health authorities, was used to select the ten organizations that met the inclusion criteria. These selected organizations were then contacted, and their representatives were emailed a request to nominate/assign team members involved in technical and operational HTA processes within their organization to complete the survey. Each team completed one survey on behalf of their respective organization.

The same inclusion criteria were applied to a second group of seven experts working in HTA policy, strategy, and systems, identified from the same HTA-associated organizations in Canada. These experts included HTA specialists, heads of HTA units, academics, policymakers, directors, and advisors. They were required to hold officially recognized senior positions in HTA or HTA-related fields at the local and/or national health system levels, including roles in HTA research, policy, management, or education. The experts with no direct or indirect role in HTA policy, strategy, and systems, and who did not meet the inclusion criteria, were excluded. Mixed sampling strategies were applied to determine the final and most relevant key informants and experts from the prepared lists. These sampling strategies included simple sampling, critical case, snowballing, convenience, and self-identified sampling [24]. These sampling strategies were also guided by the application of the pre-defined selection criteria that led to the selection of ten HTA-associated organizations and seven experts from the HTA community in Canada.

The research team, led by the Principal Investigator (MA), applied this selection approach to the recruitment of organizations and experts to ensure broad agreement and representation across sectors, organizations, and levels of research, policy, managerial, operational, and technical expertise. Given the limited size of the HTA community (including organizations and experts working in HTA), the research team set a target sample size of five to ten organizations, and one individual per organization.

### Data collection

The electronic HTA survey was designed using McGill’s RedCap cloud-based clinical software. Each of the ten HTA-associated organizations completed one survey. This was executed by sending an official invitation outlining the research objectives to the head of each organization for approval. Once approval was obtained, the research team distributed the survey via email to the organization’s nominated team leader, who guided the technical, operational, practical, and managerial staff involved in HTA in completing the survey. The research team allowed all organizations and assigned teams a two-month period to complete and return the survey and provided support to address any anticipated questions or issues related to the survey.

The IDIs were conducted via a web audio-video conferencing platform (Zoom Communications Inc., 2020), and each IDI lasted between 45 and 60 minutes. Seven in-depth interviews (IDIs) were conducted with seven experts from different sectors, disciplines, and levels, out of eleven invited. The PI, assisted by a trained co-investigator, communicated with the selected experts and coordinated and conducted the virtual IDIs. The PI and co-investigator attended the IDIs, the PI led the IDI and discussion, and the co-investigator facilitated and reported the IDI.

### Data management. analysis and sample size

All data on organization names, experts, and data from the survey and IDI were stored on a secure McGill server and were only accessible by authorized members of the research team. Survey and IDI data were stored in a secure server and then imported into two software programs for data management and analysis. Survey data was analyzed using the IBM SPSS Statistics version 29 software program. The survey data were analyzed using descriptive statistics, including frequency distribution, percentages, categories, means, and standard deviation. Comparisons were made between organizations and sectors. IDI data was audio-recorded and then simultaneously translated and transcribed in English into MS Word sheets by the PI, assisted by trained co-investigators. Transcripts were imported into the software program, MAXQDA 12 (VERBI GmbH, Berlin), for qualitative data management and analysis. Transcripts were checked by the PI to ensure quality. Two coders and co-investigators constructed and validated codes in MAXQDA by classifying transcripts into IDI using a preset coding system that was derived from study objectives. To maintain consistency, a third independent reviewer resolved disagreements. Peer iterative review of themes, participant feedback, and triangulation with survey data were performed by the PI and reviewers to strengthen credibility. The methodological approach of this study was informed by similar studies [25–28]. The COREQ approach was followed for reporting the study results.

The IDI transcripts were analyzed using thematic analysis, guided by both deductive and grounded theory approaches. The research team used a study framework, developed through expert consultation and literature review, based on six HTA system pillars to ensure systematic and rigorous interpretation. The sample size was guided by a maximum variation and determined based on availability and accessibility, relevance, diversity, and representation of the essential organizations and experts involved in HTA, taking into account the practical, operational, technical, policy, and scientific considerations raised from the consultations mentioned above.

### Ethical consideration

The ethical approval from the McGill Ethics Institutional Board in Canada under the following classification (Info-Ed File Number: 21-04-009, IRB Number: A04-E13-21A) was obtained in 2021. This McGill ethical approval was renewed and obtained under the same REB File Number: 21-04-009 in June 2025. The International Ethical Standards for Biomedical Research Involving Human Persons for implementing this work were followed [29,30]. Two consents were obtained: 1) institutional administrative consent (electronic via email) provided by heads/leaders of the participating organizations by accepting the invitation sent via email to participate in the survey, and 2) both individual consents, written via email and verbal via phone, were obtained where participants accepted the interview invitation sent via email/phone. Additionally, participants were asked to give their second verbal consent before the interview began. Multiple consents were obtained to comply with ethical guidelines, ensure their voluntary participation, confidentiality, and the right to withdraw at any time, and their data would be discarded and not included in the final data set.

## Results

### Survey findings on the HTA system from a technical perspective

In this HTA system analysis, ten organizations completed and returned the survey with sector diversity. Organizations were from five major provinces of Canada: Ontario, Quebec, British Columbia, Alberta, and Saskatchewan. These organizations from the public sector were represented by 50% (n = 5), while the academic and NGO sectors were represented by 20% (n = 2). 40% (n = 4) of these organizations were academic (not-for-profit), national, local, and hospital, with 10% each, and 30% represented the classification of others. The majority of organizations were employed in a multidisciplinary field (60%), while 10% reported working in the pharmaceutical sector, 10% in economics, and 20% in other fields such as public health and epidemiology.

90% (n = 9) of the organizations reported very high/high level of understanding of the HTA concept, 100% reported clear and very high/high knowledge of the purpose of HTA, 80% (n = 8) of organizations reported very high/high HTA applicability and HTA importance to their organization and health system, and 100% reported very high/high in HTA importance to their area or sector of work and 70%, (n = 7) very high/high, whereas 10% (n = 1) reported a moderate level.

80% (n = 8) of surveyed organizations reported they were aware of having a structured and appropriate formal process in place for collecting HTA information to support health decision-making, and only 20% were unaware of this HTA process. Half of the organizations (50%, n = 5) acknowledged the presence of a central agency responsible for HTA management, referring to federal CADTH and provincial INESSS in Quebec, (30%, n = 3) indicated the absence of the HTA agency, and (20%, n = 2) indicated they did not know the answer.

Concerning the legislation, only 30% (n = 3) affirmed they had a legislative requirement for the HTA process and results to ensure compliant financing decisions and overall public health decision-making, while 30% (n = 3) said they did not, and 40% did not know.

**Fig 1:**
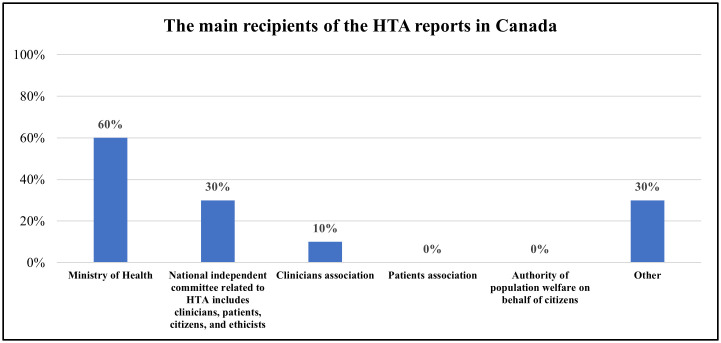
The main recipients of the HTA reports in Canada (Multiple answer question, 10 organizations provided multiple answers).

Concerning HTA recommendations and advice, two organizations declared that it is legal and must be considered, but not binding. Concerning the HTA reports, 90% (n = 9) of Canadian organizations confirmed that a national agency unit or committee produced HTA reports. Three institutional entities that produce these reports were identified as follows: HTA organizations or agencies (80%, n = 8), a national/federal committee within the Ministry of Health (MOH) (20%, n = 2), and a national unit/department within the MOH (10%, n = 1). As illustrated in Fig 1, the Ministry of Health (MOH) is the primary recipient of HTA reports, with 60% of organizations (n = 6) indicating that HTA outputs are directed to MOH decision-makers. Additionally, 30% (n = 3) reported that HTA findings are submitted to a National Independent Committee responsible for HTA-related decisions, while 10% (n = 1) identified clinician associations as key recipients. The remaining 30% of recipient organizations included procurement and financing units, academic and research institutions, professional associations, and NGOs.

**Fig 2:**
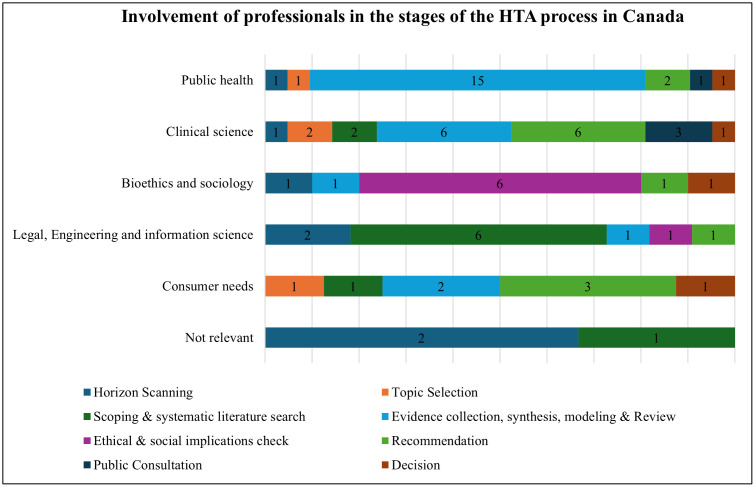
Involvement of professionals in the stages of the HTA process in Canada (types of professionals involved in the HTA process). Number of organizations responded = 10. These types include public health (epidemiologists, biostatisticians/statistician, economists/ health economists, public health professional), clinical science (medical doctor, nurse, pharmacist, health professional organization), bioethics and sociology (sociologist, ethicist), legal, engineering and information science (biomedical and /or clinical engineer, lawyer, librarian/information specialist), and consumer needs (civil society representative, patients representative).

Fig 2 demonstrates the involvement of Canadian professionals of the surveyed organizations, in numbers, in the stages of the HTA process, including report preparation, judgment assessment, appraisal, and decisions. Professionals of public health, clinical science, and customer needs were actively involved in two stages: evidence collection, synthesis, modeling, review, and recommendation. Professionals of clinical intervention (clinical subject-matter experts involved in the HTA process), legal, engineering, and information science were only involved in the stage of scoping and systematic literature search, and professionals of bioethics and sociology were involved in the stage of ethical and social implications check. More than two-thirds of the organizations in Canada (67%) believed HTA evaluations were not done internally within their own organization, but rather were carried out by external entities, either other Canadian organizations or international bodies.

Most organizations in the current survey (80%, n = 8) declared that those involved in preparing HTA reports declared conflicts of interest. 9 organizations out of 10 reported that findings of HTA reports were published in Canada by institution websites (80%, n = 8), online public platforms (50%, n = 5), or internal journals (newsletters, bulletins, digests, intranet posts, policy briefs, and reports), (10%, n = 1). Similarly, the policy outcomes based on HTA reports were also publicly available to more than half of the organizations (60%, n = 6). Sixty percent of Canadian organizations reported that civil society can give feedback on recommendations of an HTA report, and 60% (n = 6) also declared that stakeholders, including the community, can review a draft version of the assessment before the report is finalized.

**Fig 3:**
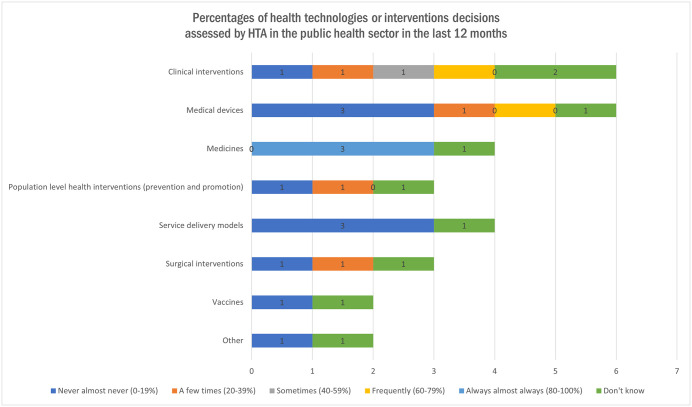
Percentages of Organizations requesting an assessment in Canada in the last 12 months (Multiple answer question, Organization responded = 10).

All the organizations in the survey declared that there was sustainable funding allocated to HTA, 70% (n = 7) by the entire federal/national and provincial government, 20% (n = 2) by the main federal/national government only, with some private contribution, and 20% (n = 2) by other funds. Organizations were asked to evaluate how many health technologies or intervention decisions had been assessed by HTA in the public health sector in the 12 months before the administration of this survey in Canada, as illustrated in Fig 3. Among the ten organizations, three reported that between 80% and 100% of medicines had been assessed through HTA processes. Other types of health technologies or interventions were evaluated less frequently or received less attention within the HTA process compared to those that were more commonly assessed.

**Fig 4:**
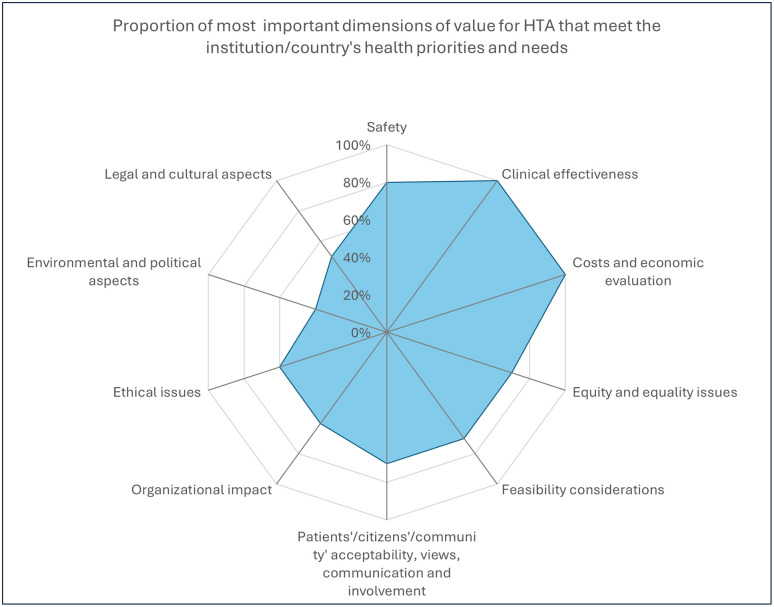
Proportion of most important dimensions of value for HTA that meet your institution/county’s health priorities and needs (Multiple answer question, Organizations responded = 10).

Fig 4 presents the most important dimensions of value for HTA meeting institutions or a country’s health priorities and needs. Most important dimensions of value were clinical effectiveness and costs and economic evaluation (Cost-effective analysis, budget analysis, utilization, unit cost, indirect cost, outcomes) (100% each, n = 10). Safety came in second with 80% (n = 8), then equity and equality issues, feasibility considerations, and Patients’/citizens’/community’ acceptability, views, communication, and involvement with 70% (n = 7).

**Fig 5:**
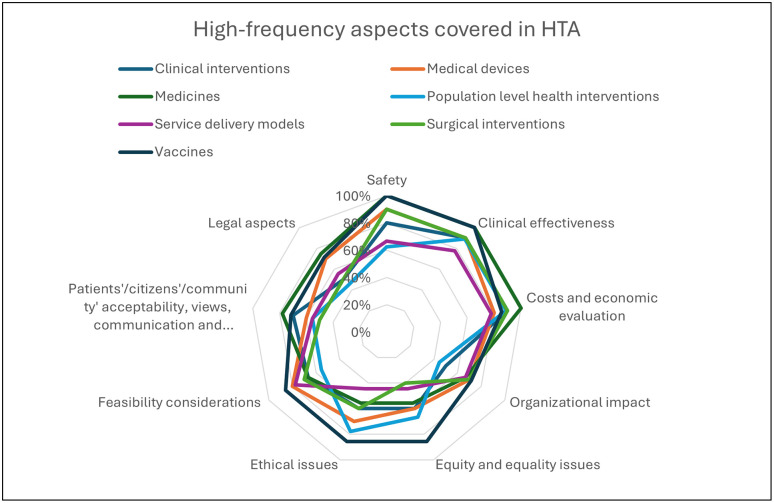
High-frequency aspects covered in HTA in Canada (Multiple answer question, Organizations responded = 10).

The frequency of covering different aspects of HTA by type of technology in Canada is presented in Fig 5. Safety, clinical effectiveness costs, and economic evaluation were the most common aspects covered in HTA in all types of technologies.

**Fig 6:**
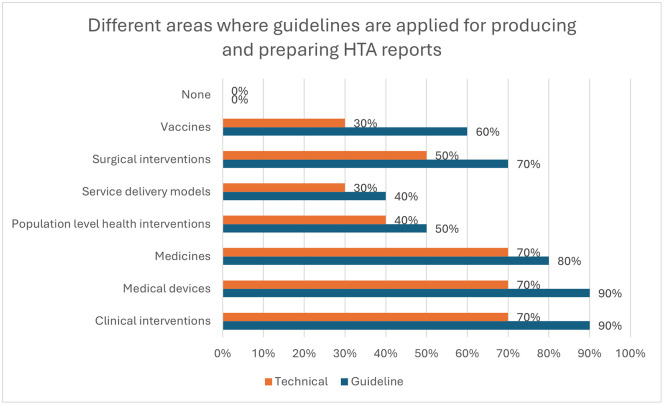
Different areas where guidelines are applied for producing and preparing HTA reports (Multiple answer questions, Organizations responded = 10).

Fig 6 shows the different areas where guidelines are applied for producing and preparing HTA reports, and where technical practices or procedures are applied for submitting HTA reports and mechanisms for communicating them. Guidelines are mainly used in clinical interventions and medical devices (90% each, n = 9), followed by medicines (80%, n = 8) and surgical interventions (70%, n = 7). The results were similar for technical practices with clinical interventions, medical devices, and medicines (70% each, n = 7), followed by surgical interventions (50%, n = 5).

**Fig 7:**
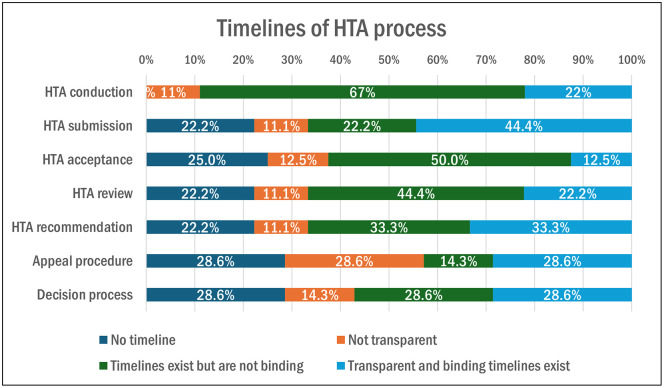
Timelines of the HTA process in Canada (Multiple answer question, Organizations responded = 10).

Fig 7 shows the steps of the HTA process for which timelines did or did not exist, whether they were transparent, or whether they were binding. All the Canadian organizations revealed they have a formal process by which information is gathered to support decision-making on new devices, drugs, or vaccines.

**Fig 8:**
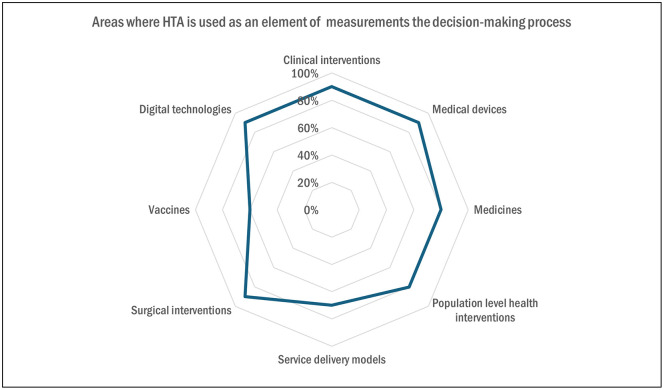
Areas where HTA is used as an element of measurement in the decision-making process (Multiple answer question, Respondents = 10).

The areas where HTA was used as an element of measurement in the decision-making process were: clinical interventions, medical devices, surgical interventions, and digital technologies (90% each, n = 9), followed by medicines and population-level health interventions (80% each, n = 8), service-delivery models (70%, n = 7) and vaccines (60%, n = 6), as demonstrated in Fig 8.

The reports of HTA findings were used on a national basis by legislation for half of the organizations (50%, n = 5), and on a regional basis by 20% (n = 2) of the organizations. Also, just under a third of them reported that reports were used on a professional (10%, n = 1), institutional entity (10%, n = 1), or institutional and national basis (10%, n = 1). The organization making the decision relied partially on the conclusions of the assessment for 50% (n = 5) of the organizations, while 20% (n = 2) reported that it was complete, and 30% (n = 3) reported that HTA was only one element of informing the decision.

**Fig 9:**
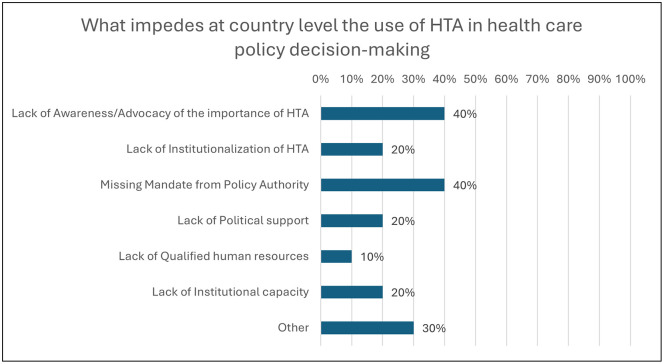
What impedes the country level in health care policy decision-making (Multiple answer question, Responses 18, Organizations responded = 10).

**Fig 10:**
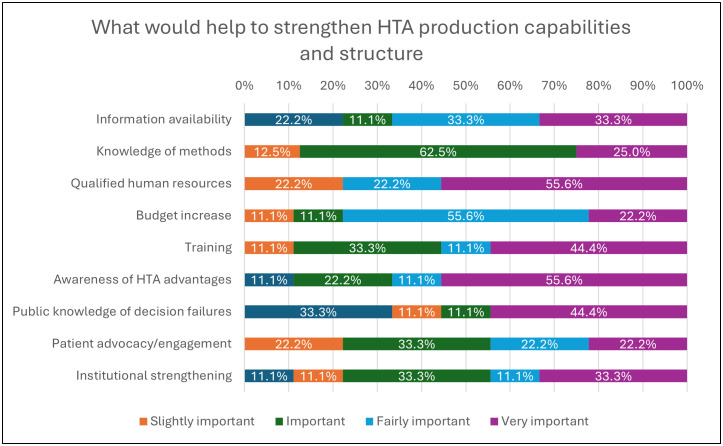
What would help to strengthen HTA production capabilities and structure (Multiple answer question, Organizations responded = 10).

Fig 9 shows the perceptions of surveyed organizations on what impedes the use of HTA in health care policy decision-making at the country level. Forty percent of organizations declared that the principal reasons were still the lack of awareness/literacy and advocacy of the importance of HTA and the missing mandate set by a policy authority. Organizations were also asked to indicate the importance of factors that would help strengthen HTA production capacity and structural arrangements. As demonstrated in Fig 10, the majority reported that qualified human resources and more awareness/literacy of HTA advantages were very important (55.6% each), and the budget increase was fairly important (55.6%).

**Fig 11:**
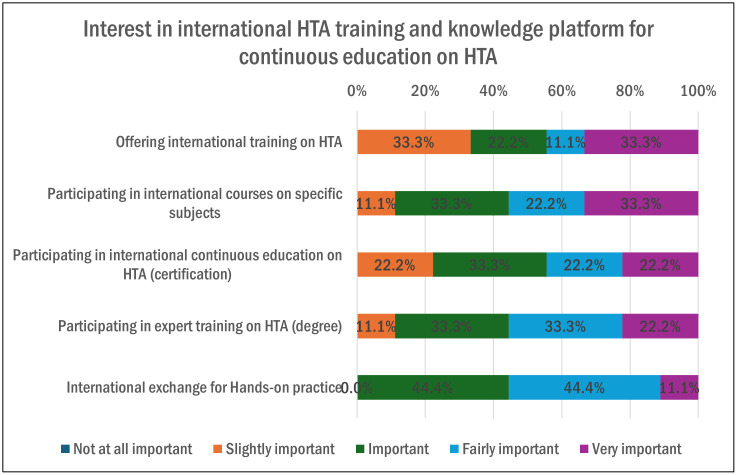
Interest in International HTA training and knowledge platform (Multiple answer question, Organizations responded = 10).

The last section is related to the academic and training programs supporting HTA capacity building in Canada. Strengthening these programs is essential, and findings highlighted several feasible options to strengthen it through courses, seminars, or workshops (90%, n = 9), higher education and master’s (80%, n = 8), and internal staff training sessions or workshops (60%, n = 6). The Canadian organizations showed some interest in international HTA training and knowledge platforms for continuous education on HTA, as illustrated in Fig 11.

### Qualitative evidence on the HTA system: Policy perspectives

This system thinking and analysis study examined the policy perspectives of seven experts, four of whom were from the public sector and three academics who were interviewed regarding HTA in Canada. The qualitative evidence analysis presented below is structured into thematic areas: common understanding and perceived importance of HTA, anticipated benefits and impacts, stewardship and management, resources and capacity supporting the HTA process, implementation of HTA, and interests and impediments to building capacity.

#### Common understanding and perceived importance of HTA.

A key area of exploration of this study was the experts’ understanding and perspectives of HTA. Most experts demonstrated a comprehensive understanding of HTA principles and processes. One public sector expert defined it as: “*A multi-disciplinary process that uses explicit methods to determine the value of our technology at different points in the life cycle. The purpose is to inform decision-making to promote an equitable, efficient, and high-quality use of health technologies stemming from multiple dimensions. would say it’s a rigorous process to gather information in the literature, in data clinical data databases*” (Interviewee C1, Public Sector Expert).

Beyond specific definitions, common perspectives emerged, portraying HTA as a robust, multi-disciplinary, and multi-level decision-making process. This process explores the uses and benefits of technology across the lifespan and drives healthcare decisions considering clinical, cost, socioeconomic, ethical, and legal aspects. Experts also described the HTA situation in Canada, acknowledging government support, infrastructure, and specialized bodies and departments in some provinces. These efforts were aimed at improving health outcomes and optimizing the allocation of healthcare resources.

In terms of perceived importance, experts consistently stressed that HTA is indispensable to evidence-informed decision-making in Canada. Several participants noted that HTA plays a critical role in ensuring that limited healthcare resources are allocated efficiently and equitably, particularly given rising healthcare costs and growing technological complexity. Experts described HTA as a “cornerstone” for transparent and accountable policy decisions and emphasized that, without HTA, health systems risk adopting technologies that are costly, ineffective, or misaligned with population needs. Others highlighted its importance in fostering consistency across provinces and supporting long-term sustainability of the Canadian healthcare system.

#### Anticipated benefits and impacts.

Experts also discussed the perceived benefits of HTA, emphasizing its potential to generate evidence-based recommendations, enhance knowledge, improve decision-making processes, and promote transparency in healthcare. However, they also highlighted potential limitations, noting the significant time commitment and specialized personnel skills required for effective HTA implementation. Experts cautioned that despite its benefits, HTA can be resource-intensive and demand specialized expertise, which may pose challenges for timely and effective implementation.

#### Stewardship and management.

The study also investigated the governance and management structures surrounding HTA in Canada. Most interviewees expressed the view that Canada lacks a formal national HTA governing body because of decentralization and the existence of provincial-level HTA organizations. They identified entities such as the MOH and other academic bodies as governance bodies. Additionally, one of the academic experts noted the existence of HTA policy at the provincial level: “*I am not aware of a national, i.e., Canadian policy, but there is one at the provincial level*”, (Interviewee 6, Academic Sector Expert).

A related concern raised by Interviewees was the absence of mandated publishing for HTA reports, which raised concerns about potential information gaps. The relevance of recommendations within HTA reports was emphasized, as well as questions raised about the extent to which such reports are read and utilized. As one public sector HTA specialist stated, “*It’s always hard to know how much people are using their reports and things. I think it’s the recommendations that matter, and that’s why the evaluation or doing will be important, so it’s unclear how many people read them. Though most cost-effective, like all that stuff is in the report, it doesn’t make it very high-level recommendations. In addition to the recommendations, I think if more people report that, it would be nice and we just make that a lot smoother and more efficient I don’t know how often that happens, but I think to improve its integration with other decision-making processes would be important and the use of HTA as a resource to support other decisions*” (Interviewee C2, Public Sector Expert).

#### Resources and capacity supporting the HTA process.

In terms of resources and capacity supporting HTA processes in Canada, interviewees indicated that international HTA standards/guidelines are widely adopted, but there are few national guidelines tailored to the Canadian context, with the existence of limited national guidelines aligned with the context of Canada. However, due to the country’s decentralized system, they were unable to identify a set of unified national guidelines. Instead, participants mentioned organizations like the US Census Bureau, Cochrane, and the WHO as sources of guidance and methodologies.

Experts described Canada’s HTA capacity as generally well-established but variable across provinces and institutions. Organizationally, HTA units operate within formal structures and legal mandates, with defined policies, procedures, and multidisciplinary teams responsible for evidence synthesis, economic modeling, public engagement, and qualitative and quantitative analysis. While some provinces, such as Québec, have strong infrastructure and sufficient funding, others face challenges in human, technical, and intellectual resources, including limited expertise in emerging areas like artificial intelligence and digital health. Participants highlighted difficulties in integrating patient perspectives and administrative databases, as well as the need for leadership that can anticipate innovation. At the individual level, teams are composed of PhD- and master’s-trained experts, but gaps remain in specialized skills, experience, and training. Overall, while progress has been made, experts emphasized the need for ongoing capacity building and resource development to ensure that HTA processes can keep pace with evolving technologies and health system demands.

### The implementation process of HTA

The interview responses regarding the application of HTA in health sector decision-making revealed mixed perspectives. Although many experts concurred that treatment and healthcare management recommendations undergo an HTA process, one expert indicated that capacity and budget limitations still pose challenges to the systematic use of HTA in decision-making: “*I think Canada has pushed this agenda of using HTA as a decision-making tool…*” (Interviewee C4, Academic Sector Expert).

#### Opportunities and impediments to building capacity.

Finally, the study explored opportunities for improvement and capacity building in the Canadian HTA landscape. Experts highlighted potential opportunities for improved coordination and alignment at the national level, specifically concerning methodologies, budgets, capacity development, and training initiatives. They recognized the need for increased awareness and literacy of HTA processes, both locally and nationally. Furthermore, experts identified the importance of dedicated funding streams, sufficient human resources, effective communication strategies, and strengthened collaboration between regulatory bodies and communities to enhance HTA capacity and impact.

## Discussion

HTA plays a pivotal role in high-income countries by contributing to decision-making processes and fostering the development of high-quality healthcare systems [2]. This study adopted a systems thinking and analysis approach to comprehensively analyze and understand the HTA landscape in Canada, recognizing the multitude of stakeholders and organizations involved. As a unique and in-depth exploration, this comprehensive system analysis of the HTA system reveals a nuanced interplay of both systematic strengths and persistent challenges. The study achieved its goals by supporting that the Canadian HTA system and its organizations had a high degree of comprehension and acknowledgment of HTA’ goal and relevance, as well as evidence of organized procedures and steady funding for HTA. Decentralization issues, a lack of uniform norms, and a lack of awareness/literacy and advocacy of the importance of HTA, however, brought to light important areas for enhancing national coordination and capacity building.

Survey findings complement the qualitative themes, confirming the high knowledge and perceived relevance of HTA concepts across sectors. Governance and stewardship emerged as a theme, with half of the organizations acknowledging a central HTA agency, while others indicated provincial or unknown structures, reflecting the decentralized system. Resources, capacity, and implementation were additional themes, showing that structured HTA processes exist, yet limitations in timelines, systematic application, and stakeholder literacy hinder full utilization. The survey also supported the theme of HTA benefits and impacts, with organizations reporting the use of HTA reports in decision-making for clinical interventions, medical devices, medicines, and population-level interventions. This indicates a strong culture and widespread practice of using HTA evidence to support decision-making across various HTA domains among most Canadian organizations. Finally, both data sources underscored the need for capacity building and training, highlighting the importance of national coordination, dedicated funding, and workforce development to strengthen HTA production, report use, and policy influence. The foundational awareness supports its effective application in decision-making and aligns with global recognition of HTA as a fundamental tool for informed healthcare policy [31]. This awareness is further promoted through stakeholder engagement in national and global HTA processes and practices. Furthermore, these findings support the broader goal of achieving equitable and efficient health systems, consistent with practices implemented in other high-income countries [3].

Most stakeholders indicated awareness of existing structured processes, while only a few non-state organizations—such as NGOs and hospitals—reported limited awareness, likely due to limited involvement at the national level or insufficient access to information. The stakeholders emphasized the system benefits from structured processes for collecting HTA information, reflecting a commitment to systematic management of HTA processes. This aligns with recent research highlighting the importance of structured approaches in HTA. A 2021 study by Oortwijn et al. emphasized the need for robust methodological frameworks in HTA to ensure consistency and reliability in assessments [32]. The HTA system in Canada requires the adoption and application of comprehensive and structured approaches that align the healthcare organizations at the provincial and federal levels. Conducting a national HTA based on agreed ethical, clinical, economic, and clinical criteria, improving adoption pathways: e.g., HTA recommendations published within 6 months, and establishing mechanisms to track the proper implementation of HTA processes and recommendations, can be good examples of these robust HTA methodological frameworks. Stakeholders indicated high levels of literacy and understanding of HTA concepts, recognizing its purpose, applicability, and importance, with most respondents reporting strong knowledge. This positive and strong perception of stakeholders reinforces HTA’s role as a rigorous, multi-disciplinary process that integrates clinical, economic, socioeconomic, ethical, and legal considerations across the technology lifecycle.

Importantly, the current supportive infrastructure and government involvement in Canada were cited as essential enablers for achieving better health outcomes and optimizing resource allocation. Nevertheless, the challenge remains in ensuring the consistent application of these structures across Canada’s varied regional and sectoral contexts. While the federal government demonstrates a strong commitment to HTA, there is a clear need for ongoing system refinement to accommodate emerging healthcare innovations and to address regional implementation disparities through addressing decentralization, standardizing guidelines, and sustainable funding. Decentralization in Canada’s HTA system can be addressed through national coordination, national standardized guidelines alignment and contextualization, collaborative provincial implementation mechanisms, and strengthened legislative and regulatory oversight to ensure consistent and equitable application across regions. Despite challenges inherent in a decentralized healthcare system, Italy has addressed regional disparities through structural reforms and investments in digital and care networks, aiming to enhance equity, resilience, and access across regions [33].

The study further reveals that HTA activities in Canada are underpinned by sustainable funding mechanisms predominantly sourced from government allocations. HTA reports are made accessible through institutional websites and public platforms, enhancing transparency and accountability. This aligns with global best practices, where transparency in HTA processes has been shown to improve stakeholder engagement and trust in healthcare policies [34]. A study by Suharlim et al. (2021) emphasizes that as healthcare technologies advance, HTA systems must be equipped with adequate financial and human resources to keep pace with innovation and maintain their relevance [35]. This raises the question of whether Canada’s current funding mechanisms are flexible enough to accommodate the growing complexity through inter-provincial and inter-organizational cooperation and exchanging the necessary resources (human and financial) and volume of HTA demands.

Another notable finding is the strong involvement of multidisciplinary professionals across various stages of the HTA process in Canada. This diversity of expertise is vital for ensuring comprehensive assessments that consider clinical, economic, ethical, and social dimensions. This finding aligns with a recent study highlighting Canada’s extensive international leadership and collaborations shows highly diverse professional involvement in HTA [36].

The study also highlights the critical importance of key dimensions such as clinical effectiveness and economic evaluation, which are central to aligning HTA with broader healthcare goals. This finding is consistent with Chen (2022), who emphasized that clinical effectiveness and economic evaluation are central dimensions of HTA, ensuring that recommendations are evidence-based and support cost containment and improved patient outcomes within constrained healthcare budgets [37]. Effective integration of these dimensions ensures that HTA recommendations are not only evidence-based but also aligned with health system priorities, such as cost containment and improved patient outcomes. This is particularly important as healthcare systems globally face mounting pressure to deliver high-quality care within constrained budgets. However, there are gaps such as decentralization, adaptation to growing complexity and variability in application, which have emerged due to the decentralized system in Canada.

HTA’s pivotal role in reimbursement decisions and clinical guidance further underscores its impact on healthcare policy. Structured HTA processes offer a reliable and evidence-based framework for evaluating healthcare technologies, thereby enhancing the quality of policymaking and resource allocation. However, the translation of HTA assessments to policy is not always systematic, especially when resources and funding are not even. There is a need for more consistent procedures, proper resources, and continued stakeholder participation.

Capacity and resource constraints, including budget limitations and gaps in technical expertise, further complicate the landscape. These limitations hinder the ability of provinces to conduct thorough and timely assessments, ultimately affecting the integration of HTA into policy decisions. A 2019 study by Loblova et al. highlighted similar capacity challenges in other countries, emphasizing the need for continuous investment in HTA infrastructure and workforce development to sustain effectiveness and credibility [38]. The lack of dedicated trained personnel in HTA not only limits the scope of assessments but also affects the overall quality and timeliness of HTA outputs. This offers a great opportunity to strengthen the existing HTA training/education to be well institutionalized into the academic and healthcare organizations through formal and non-formal continuous programs with good allocation of HTA expertise in the learning process. Australia, Singapore, and New Zealand have well-established HTA programs and collaborate through networks like HTAsiaLink to strengthen HTA capabilities in the region [39]. Also, other leading countries in HTA operate federally coordinated programs, such as Switzerland, where HTA is governed under Article 32 of the Federal Health Insurance Act (KVG; SR 832.10), and Brazil, where HTA programs are coordinated through the National Committee for the Incorporation of Technologies in the Unified Health System (CONITEC) [15,16].

Moreover, the absence of unified national guidelines, despite references to international HTA standards, introduces variability in assessment methodologies. This lack of harmonization can lead to disparities in the adoption of healthcare technology and reimbursement decisions across provinces [4]. This is a shared challenge among decentralized systems. A comparative policy analysis of HTA reforms across multiple countries identified that decentralized health systems face similar challenges in aligning HTA processes, underscoring the importance of national coordination and standardization to achieve equitable outcomes [40]. Without a concerted effort to establish common frameworks, inconsistencies in evaluation criteria and decision-making processes will likely persist. Establishing national HTA evaluation criteria and guidance, alongside provincial mechanisms for applying real-world evidence, can facilitate high-quality and transparent HTA decision-making in Canada [41]. This framework should be developed collaboratively by CADTH and Health Canada, in coordination with provincial HTA units, to ensure consistent national standards and guidance while allowing provinces to implement context-specific mechanisms for generating and using real-world evidence.

Legislative and regulatory support could play a vital role in strengthening HTA governance and streamlining operations across regions. A 2013 study by Hailey et al. highlighted the positive impact of stakeholder engagement and public communication on the uptake of HTA recommendations [42]. Enhanced national oversight, including legislative frameworks, would support the development of standard guidelines, improve cross-provincial collaboration, and ensure that HTA outputs are translated more effectively into policy action. This aligns with broader HTA system analyses, which have consistently emphasized the benefits of evidence-based recommendations, improved transparency, and informed decision-making. However, these benefits can only be fully realized if systemic barriers—such as time constraints and personnel shortages—are adequately addressed. This could be addressed by equitable and proper mobilization of resources across provinces and training more healthcare policymakers, managers, and professionals to support the decision-making. The findings suggest that the federal government should play a stronger oversight role in HTA, using aligned and contextualized legislative frameworks, national guidelines, and coordinated resource mobilization to enhance cross-provincial consistency, stakeholder engagement, and effective translation of HTA outputs into policy.

In addition, the study identifies several opportunities for system enhancement and to address gaps, including improved national coordination, targeted capacity-building initiatives, and increased awareness and advocacy efforts. Investing in training programs, workshops, and higher education in HTA could address existing skill gaps and develop a more robust workforce capable of meeting evolving healthcare technology assessment demands. These suggestions are consistent with recommendations from a 2017 global survey by Oortwijn et al., which emphasized the importance of collaborative networks and knowledge-sharing platforms in strengthening HTA systems [34].

The effective integration of HTA findings into institutional and national decision-making remains a persistent challenge. Strengthening the link between HTA outputs and policy implementation requires collaborative effort across the spectrum of stakeholders, including government bodies, healthcare providers, academic institutions, and industry actors. One study concluded that it is essential for HTA regulators to maintain continuous and public communication with various stakeholders regarding HTA decisions, as this fosters trust and enhances confidence in the regulators [36]. For example, in England, stakeholder participation in HTA, which is mostly run by the National Institute for Health and Care Excellence (NICE), has a big impact [43]. The benefits of HTA – such as improved transparency, better-informed decisions, and cost-effective healthcare – are only fully recognized when these systematic barriers are addressed through strategic, sustained action.

This study provides a foundational benchmark for understanding the HTA landscape in Canada and offers valuable mixed-method tools for systematic evaluation. However, a more expanded sampling that includes more organizations from other provinces is recommended to capture greater nationwide HTA experiences and to draw federal policy recommendations. Information on the five major Canadian provinces included in this study—Ontario, Quebec, British Columbia, Alberta, and Saskatchewan—is presented in the first section of the results. It is recommended that future research consider the remaining provinces to capture a more complete picture. Consequently, this sample may not reflect the full range of experiences across Canada, and the response proportions may not be generalizable to all provinces.

## Conclusion

In conclusion, this study, which adopts a systems thinking and analysis approach, provides valuable insight into the current state of the National HTA System in Canada, highlighting both the strengths and challenges that shape its policy, governance, and processes’ effectiveness. Stakeholders’ positive perceptions of HTA and the presence of structured processes demonstrate a solid foundation for leveraging HTA to inform healthcare policy and improve health outcomes. The study also identifies key strengths of the Canadian HTA system, including robust legislative and policy frameworks, established institutional structures, and the availability of resources. However, further action is needed, including the establishment of inclusive and unified federal and interprovincial HTA frameworks and strategies, improved adoption pathways of HTA recommendations, adaptation of HTA methodologies and standards to enable more flexible and timely evaluations, and increased investment in HTA capacity building and education. These actions can support creating an institutional decision-making process backed by a participatory HTA system. This system addresses the regional disparities and varying strategies, methodologies, and approaches. This vision is essential to unlocking the full potential of HTA in Canada, and it can be driven by CADTH in a close partnership with the public, private, community, and academic sectors.

## Supporting information

S1 Text Questionnaire Form. (PDF)

S2 Text Interview Guide. (DOCX)

S1 Checklist COREQ Checklist table. (DOCX)
